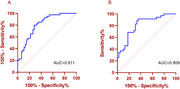# Delayed niacin skin flush response and cognitive impairment in Late‐Life Depression

**DOI:** 10.1002/alz70857_097465

**Published:** 2025-12-24

**Authors:** Yan Chen, You Wu, Dandan Wang, Yang Yang, Qianqian Guo, Qi Qiu, Chunlin Wan, Xia Li

**Affiliations:** ^1^ Shanghai Jiao Tong University School of Medicine, Shanghai, Shanghai, China; ^2^ Longhua Hospital Shanghai University of Traditional Chinese Medicine, Shanghai, 200030, China; ^3^ Shanghai Jiao Tong University, Shanghai, Shanghai, China; ^4^ Shanghai mental center, Shanghai, Shanghai, China; ^5^ shanghai mental health centre, shanghai, shanghai, China; ^6^ Shanghai Mental Health Center, Shanghai Jiao Tong University School of Medicine, Shanghai, China

## Abstract

**Background:**

Late‐life depression (LLD) with cognitive impairment (CI), as a potential subtype of LLD, carries an elevated risk of progressing to Alzheimer's disease (AD), yet the underlying mechanisms remain elusive. While anomalies in niacin skin flushing response (NSFR) have been observed in various neuropsychiatric disorders, there is a paucity of research examining these phenomena in LLD patients. This study aims to elucidate the potential value of the NSFR in predicting CI in LLD patients.

**Method:**

This study included 86 patients of LLD (46 LLD with CI and 40 LLD without CI), 20 AD and 32 Healthy Controls (HCs). Cognitive functions were estimated through the Chinese version of Montreal Cognitive Assessment (MoCA). NSFR tests were conducted with a modified method. LogEC_50_ is utilized to indicate the rate of NSFR. MoCA was retested after six months of treatment. Multivariate analysis of variance (MANOVA) was conducted with demographic variables that differed statistically among the groups to assess differences in NSFR and clinical indexes among groups. Logistic regression models based on NSFR were constructed, and receiver‐operating characteristic (ROC) curve analysis was calculated to evaluate the performance of models.

**Result:**

The LogEC_50_ levels were significantly elevated in LLD with CI group compared to both the LLD without CI (*p* <0.05) and HCs (*p* <0.05). However, no significant differences were observed between the LLD without CI and the HCs group, nor between the LLD with CI and AD. ROC analysis demonstrated that Log EC_50_ can effectively distinguish between LLD with CI and LLD without CI. Six‐month follow‐up data revealed that the baseline LogEC_50_ can also effectively predict cognitive outcomes in LLD.

**Conclusion:**

LLD with CI exhibited a delayed NSFR. Delayed NSFR proved effective in distinguishing cognitive impairment in LLD, suggesting that NSFR could serve as a potential biomarker for LLD with CI. Furthermore, in patients with LLD, a delayed NSFR at baseline predicts poorer cognitive outcomes. These insights open new avenues for research into the mechanisms underlying CI in LLD and offer fresh perspectives on potential therapeutic targets.